# Antioxidant activity and short-chain fatty acid production of lactic acid bacteria isolated from Korean individuals and fermented foods

**DOI:** 10.1007/s13205-021-02767-y

**Published:** 2021-04-15

**Authors:** Chang-Ho Kang, Jin-Seong Kim, Hye Min Park, Seonyoung Kim, Nam-Soo Paek

**Affiliations:** MEDIOGEN Co. Ltd., Biovalley 1-ro, Jecheon-si, Chungcheongbuk-do, Incheon, 27136 Korea

**Keywords:** Probiotics, Antioxidant, Anti-inflammation, Short-chain fatty acid

## Abstract

Compounds of the cell walls of heat-killed lactic acid bacteria show immunomodulatory properties which boost immunological systems, and are used ad postbiotics (paraprobiotics). In this study, we used 17 different heat-killed isolates as postbiotics and evaluated their anti-inflammatory potential on the expression of proinflammatory mediators and cellular signaling pathways of murine macrophage, RAW 264.7 cells. *Bifidobacterium bifidum* MG731 showed the high 2,2-diphenyl-1-picrylhydrazyl (DPPH) free radical scavenging activity (90.6%), followed by *Bifidobacterium lactis* MG741 (59.6%). The *Bi. lactis* MG741 showed the high ABTS free radical scavenging activity (99.5%), followed by *Lactobacillus plantarum* MG989 (98.9%), *Lactobacillus salivarius* MG242 (97.1%), and *Bi. bifidum* MG731 (96.1%). In addition, *Bi. bifidum* MG731 showed the lowest nitric oxide production (4.28 µM), followed by *B. lactis* MG741 (10.80 µM), *L. salivarius* MG242 (14.60 µM), and *L. plantarum* MG989 (19.60 µM). The selected strains showed a decreased nitric oxide production via downregulation of inducible nitric oxide synthase and cyclooxygenase 2, which were upregulated via LPS-stimulated RAW 264.7 macrophages. Short-chain fatty acids (SCFA) including acetic, propionic, and butyric acid were produced by four strains. The *Bi. bifidum* MG731 showed total SCFAs production (4998.6 µg/g), *Bi. lactis* MG741 (2613.9 µg/g), *L. salivarius* MG242 (1456.1 µg/g), and *L. plantarum* MG989 (630.2 µg/g). These results indicated that the various selected strains may possess an anti-inflammatory potential and provide a molecular basis for the development of functional probiotics.

## Introduction

Probiotics are defined as “living microorganisms that have health benefits beyond inherent basic nutrition” when consumed in certain quantities (Klein et al. 1998; Rendondo et al. 2019). According to the current definition, a probiotic must contain viable cells; therefore, it is not applicable to dead bacterial cells or cell components. Postbiotics are soluble factors (products or metabolic by-products) either secreted by live bacteria (such as probiotic or non-probiotic), or released after bacterial lysis, that may be beneficial to the host (Aguilar-Toalá et al. 2018). The mechanisms implicated in most bioactivities of postbiotics are not fully understood; scientific evidence supports that postbiotics possess diverse functional/bioactive properties such as antimicrobial, antioxidant, and immunomodulatory activities via direct (interaction with the intestinal microbiota or immune cells) or indirect (outside the gastrointestinal tract, in the immune system and other organs) pathways (Aguilar-Toalá et al. 2018; de Almada et al. 2016; Sharma and Shukla 2016).

Inflammation is a complex response of the vascular tissues to harmful stimuli, such as pathogens, damaged cells, or irritants. It is mediated by a variety of signaling molecules produced by macrophages, monocytes, and mast cells. The stimulus is persistent in chronically inflamed tissues; therefore, recruitment of the monocytes is maintained and existing macrophages are tethered in place. Macrophages play an important role in the innate immunity as they are one of the first cells to respond to microbial infection. They can kill pathogens directly via phagocytosis and indirectly via the secretion of proinflammatory cytokines such as tumor necrosis factor alpha (TNF-α), interleukin 1 beta (IL-1β), and interleukin 6 (IL-6) (Stuehr et al. 1991; Kazemi et al. 2019), as well as excess amounts of mediators such as nitric oxide (NO), and prostanoids in response to lipopolysaccharide (LPS) stimulation.

Short-chain fatty acids (SCFAs) such as butyrate, propionate, and acetate are produced via fermentation of indigestible carbohydrates by intestinal anaerobic commensal bacteria such as *Clostridia* species; they impact both nonimmune as well as immune intestinal cells (Koh et al. 2016). SCFAs play an important role in innate immunity as they are histone deacetylase (HDAC) inhibitors with anti-inflammatory effects (Willemsen et al. 2003). Additionally, SCFAs play a role in the adaptive intestinal immunity (Willemsen et al. 2003). It was shown that the administration of butyrate, acetate, and propionate to germ-free mice increases the expression of anti-inflammatory interleukin 10 (IL-10) producing Foxp3-expressing regulatory T cells (Tregs) via HDAC inhibition (Smith et al. 2013). Moreover, butyrate increases interleukin 18 (IL-18) expression in epithelial cells, increases the IL-10 expression in dendritic cells and macrophages, and enables them to induce the differentiation of Tregs, thereby conferring protection against colitis (Singh et al. 2014). Therefore, SCFAs affect both nonimmune as well as immune intestinal cells and modulate intestinal homeostasis.

Although these studies have reported the functional activities of various lactic acid bacterial strains, lactic acid bacteria strains against LPS-induced inflammation have not yet been reported. Therefore, we aimed to evaluate the anti-inflammatory potential of 17 lactic acid bacterial strains. The effects of 17 heat-killed lactic acid bacteria on the expression of proinflammatory mediators and cellular signaling pathways were investigated in LPS-induced murine macrophage, RAW 264.7 cells.

## Materials and methods

### Sample preparation

In this study, 17 strains were isolated from Korean individuals and fermented food products (Kim et al. 2020). The selected strains were identified by the 16S rRNA gene sequencing method (SolGent Co., Ltd. Korea). The strains were cultured in de Man, Rogosa, and Sharpe (MRS) broth (BD Biosciences, Frankin Lakes, NJ, USA) at 37 ℃ and the strains were stored at deep freezer (−80℃) in 20% (v/v) glycerol. To evaluate the anti-inflammatory potential of these strains, selected strains that were cultivated overnight were heat-killed at 90 ℃ for 30 min. Following centrifugation (12,000 × g, 5 min), the cell pellets were rinsed thrice with phosphate-buffered saline (PBS) and suspended in Dulbecco’s modified Eagle media (DMEM) (BD Biosciences) to obtain concentrations of 5 × 10^8^ cells/mL by adjusting the solution based on absorbance at 600 nm (OD_600_). The mixture samples comprised mixed cultures of the 17 strains in a same ratio, respectively, and freeze-dried.

### In vitro antioxidation properties of the selected strains

The 2,2-diphenyl-1-picrylhydrazyl (DPPH) radical scavenging assay was performed according to Blois (1958) with minor modifications. Briefly, culture of the selected strains were adjusted to an OD_600_ of approximately 1.0 using PBS (pH 7.4) and were added to 0.05 mM DPPH solution (1:2 v/v) and mixed well. Next, the mixtures were left to stand at room temperature for 30 min in the dark. The control reaction involved ethanol added to DPPH solution. The absorbance of each mixture was measured at 517 nm. Each sample assay was performed in triplicate. The results were compared with those of ascorbic acid (10 μg/mL), and the antioxidant activity was calculated using the following formula: Scavenging effect (%) = (Ac-As)/Ac × 100, where As is the absorbance of the test sample, and Ac is the absorbance of the control at 517 nm.

The scavenging activity of the (2,2′-azino-bis(3-ethylbenzothiazoline-6-sulfonic acid)) (ABTS) radical was measured according to Re et al. (1999). Briefly, the radical cation was prepared by mixing 7 mM of ABTS with 2.45 mM potassium persulfate (1:1 v/v) and leaving the mixture at room temperature in the dark for 24 h. Next, 50 μL of the selected strain sample and 100 μL of ABTS solution were mixed and incubated for 10 min at room temperature. The absorbance of the mixture was measured at 734 nm. Each sample assay was performed in triplicate, and the scavenging rate was calculated as follows: Scavenging rate (%) = (Ac-As)/Ac × 100, where As is the absorbance of the test sample, and Ac is the absorbance of the control at 734 nm.

### Cell culturing of RAW 264.7 cells

The murine macrophage RAW 264.7 cell line was obtained from the Korean Cell Line Bank (KCLB, Korea) and incubated in DMEM (BD Biosciences) supplemented with 10% fetal bovine serum (FBS; Gibco) and 1% penicillin/streptomycin at 37 ℃ in 5% CO_2_. The cells were sub-cultured and plated at 80‒90% confluency.

### NO production

RAW 264.7 macrophage cells were grown at 37 ℃ and 5% CO_2_ in a fully humidified atmosphere and sub-cultured every 3 days to 95% confluency. For routine subcultures, DMEM was supplemented with 10% FBS, penicillin (100 units/mL), and streptomycin (100 μg/mL). NO formation was detected based on the accumulation of nitrite, an indicator of NO synthesis, in the culture medium via the Griess reaction (Lyons et al. 1992). RAW 264.7 cells were plated at 2 × 10^5^ cells/well in a 96-well plate and stimulated with 1 μg/mL LPS, followed by the addition of isolated bacterial strains (10^9^ cells/well). After 24 h of incubation, NO concentration was determined by measuring the amount of nitrite in the cell culture supernatant using the Griess reagent. The absorbance at 550 nm wavelength was measured using the Epoch 2 microplate reader (BioTek, Winooski, VT, USA). Fresh culture medium was used as the blank control for all experiments.

3-[4,5-Dimethylthiazole-2-yl]-2,5-diphenyltetrazolium bromide (MTT; Sigma, USA) assay was performed to determine the viability of RAW 264.7 cells treated with the strains. RAW 264.7 cells were washed twice with PBS and 100 μL of MTT reagent (0.5 mg/mL) dissolved with PBS was added to each well. After 1 h of incubation, the MTT reagent was discarded and 100 μL of dimethyl sulfoxide (DMSO; Sigma, USA) was added to dissolve the formazan formed as a reactant between the MTT reagent and metabolites of live cells. The absorbance (A) was measured at 570 nm wavelength, and cytotoxicity was calculated in comparison with the result of a negative control group as follows.

$$\text{Cell viability }\left( \% \right) = \left( {{\text{A sample}/ \text{A negative control}}} \right) \times 100$$

### Semi-quantitative reverse transcription-polymerase chain reaction (RT-PCR)

RT-PCR was performed to analyze the mRNA expression of inducible nitric oxide synthase (iNOS), cyclooxygenase 2 (COX-2), and TNF-α. Total RNA was extracted from the RAW 264.7 cells using the TRI REGENT™ (Sigma Aldrich, St. Louis, MO, USA) according to the manufacturer’s instructions. iNOS, COX-2, and TNF-α primers were designed for RT-PCR. Glyceraldehydes-3-phosphate dehydrogenase (GAPDH) was used as a housekeeping gene to normalize all samples. Table 1 shows the sequences of the primer pairs used to amplify iNOS, COX-2, TNF-α, and GAPDH. RT-PCR was performed using the ONE-STEP RT-PCR PreMix kit™ (Qiagen Inc., Valencia, CA, USA) according to the manufacturer’s instructions. Each of the primers and 1 μg of the RNA template were mixed with the ONE-STEP RT-PCR PreMix™. These samples were amplified via one step RT-PCR, under the following conditions: reverse transcription reaction at 95 °C for 5 min; 30 cycles of 95 °C for 45 s, 60 °C for 45 s (COX-2 and GAPDH), and 72 °C for 1 min or 35 cycles of 95 °C for 45 s, 63 °C for 45 s (iNOS and TNF-α), and 72 °C for 1 min; and a final elongation step of 5 min at 72 °C. The levels of iNOS, COX-2, and TNF-α mRNA expression were quantitated using a densitometer and using the Quantity One software (Bio-Rad, Hercules, CA, USA).

**Table 1:** Primer sequences of the primers used for reverse transcription-polymerase chain reaction assay

Gene		Sequence
TNF-α	Sense Antisense	5′-AGCCCACGTCGTAGCAAACCACCAA-3′ 5′-AACACCCATTCCCTTCACAGAGCAAT-3′
iNOS	Sense Antisense	5′-CCCTTCCGAAGTTTCTGGCAGC-3′ 5′-GGCTGTCAGAGCCTCGTGGCTT-3′
COX-2	Sense Antisense	5′-GGAGAGACTATCAAGATAGTGATC-3′ 5′-ATGGTCAGTAGACTTTTACAGCTC-3′
GAPDH	Sense Antisense	5′-TGAAGGTCGGTGTGAACGGATTTGGC-3′ 5′-CATGTAGGCCATGAGGTCCACCAC-3′

*TNF-α* tumor necrosis factor alpha; *iNOS* inducible nitric oxide synthase; *COX-2* cyclooxygenase 2; *GAPDH* glyceraldehyde-3-phosphate dehydrogenase

### Analysis of SCFAs present in the culture medium

The SCFAs in the fermented broth were analyzed via gas chromatography–mass spectrometry (QP2020 NXW/ORP230; Shimadzu, Japan) using the headspace solid-phase microextraction method (Thitiratsakul and Anprung 2014) with minor modifications. The samples were separated using a Stabilwax-DA column (60 m × 0.32 mm × 0.25 μm, Shimadzu). Analytical conditions were as follows: oven temperature was held at 50 °C for 2 min, and raised to 100 °C at 10°C/min, 200 °C at 2 °C/min, increased to 220 °C at 20 °C/min, and maintained for 2 min; the splitless mode was used; helium was used as the carrier gas at the flow of 2 mL/min. The mass spectrometer was operated in the electron-impact mode at 65 eV. The scan range was 40–200 m/z, the scan rate was 0.2 s/scan, and the electron energy was 70 eV. The ionization source and quad temperature were at 200 °C and 150°C, respectively. Each extracted sample peak area was normalized to the initial volume of the sample following quantification. Linear regression equations for each analyte were calculated with the calibration curve of the peak area versus analyte concentrations (µmol).

### Statistical analysis

Results are expressed as the mean ± standard deviation of three experiments. The difference between groups was evaluated using the Student’s *t* test, and a *P* value of < 0.05 was considered significant.

## Results and discussion

### In vitro antioxidation properties of samples

The antioxidant activities of the 18 samples (17 strains and mixture sample) were evaluated by measuring the DPPH and ABTS radical scavenging activities. The DPPH free radical scavenging activity of the postbiotic samples ranged from 17.8 to 90.6% (Table. 2). *Bifidobacterium bifidum* MG731 showed the highest radical scavenging activity (90.6%) followed by *Bi. lactis* MG741 (59.6%). The ABTS radical scavenging activity of the samples ranged from 27.1 to 99.5% (Table. 1). *Bi. lactis* MG741 showed the highest radical scavenging activity (99.5%) followed by *Lactobacillus plantarum* MG989 (98.9%), *L. salivarius* MG242 (97.1%), and *Bi. bifidum* MG731 (96.1%). In this context, de Oliveira Coelho et al. (2019) reported that both the intracellular and extracellular contents of *Lactobacillus satsumensis, Leuconostoc mesenteroides*, and *Saccharomyces cerevisiae* showed antioxidant activity ranging from 20 to 28% of the DPPH inhibition. *L. satsumensis* and *S. cerevisiae* showed the highest intracellular and extracellular activities (approximately 28% of DPPH inhibition), respectively. Similarly, Amaretti et al. (2013) reported antioxidant activities of intracellular postbiotics from 7 *Bifidobacterium*, 11 *Lactobacillus*, 6 *Lactococcus*, and 10 *Streptococcus thermophilus* strains. Afify et al. (2012) reported that *L. reuteri* showed ABTS radical scavenging effects. Lin and Yen (1999) evaluated the inhibitory effect of *Bifidobacterium longum*, and Kim et al. (2003) isolated *Bifidobacterium* species showing antioxidant activity from infant fecal samples.

**Table 2:** Radical scavenging activity of heat-killed culture samples

Isolated from	Samples	DPPH radical scavenging (%)	ABTS radical scavenging (%)
Breast milk	*L. reuteri* MG505	40.9 ± 9.2	60.9 ± 2.8
Infant feces	*Bi. longum* MG723	51.9 ± 3.7	75.2 ± 0.5
	*Bi. breve* MG729	50.2 ± 1.7	76.6 ± 0.1
	*Bi. bifidum* MG731	90.6 ± 2.3	96.1 ± 0.4
	*Bi. lactis* MG741	59.6 ± 2.1	99.5 ± 0.7
Human	*L. gasseri* MG4247	31.7 ± 2.9	62.2 ± 0.3
Human vagina	*L. salivarius* MG242	56.9 ± 6.0	97.1 ± 0.4
	*L. fermentum* MG901	22.4 ± 5.6	48.0 ± 1.8
	*L. plantarum* MG989	59.4 ± 3.8	98.9 ± 0.5
Fermented food	*L. paracasei* MG310	19.5 ± 5.0	28.2 ± 0.9
	*L. casei* MG311	19.4 ± 11.5	29.3 ± 0.7
	*L. rhamnosus* MG316	42.5 ± 10.5	49.2 ± 0.6
	*L. bulgaricus* MG515	51.7 ± 2.8	76.1 ± 8.3
	*L. helveticus* MG585	45.3 ± 4.2	84.0 ± 0.6
	*Lac. lactis* MG5125	18.4 ± 1.8	47.8 ± 1.6
	*S. thermophilus* MG5140	17.8 ± 1.6	27.1 ± 0.4
	*L. acidophilus* MG5228	55.9 ± 4.0	87.4 ± 1.4
Mixture		44.1 ± 0.8	73.6 ± 0.3

DPPH, 2,2-diphenyl-1-picrylhydrazyl; ABTS, 2,2′-azino-bis(3-ethylbenzothiazoline-6-sulfonic acid)

### Cytotoxicity and NO production

**Fig. 1:**
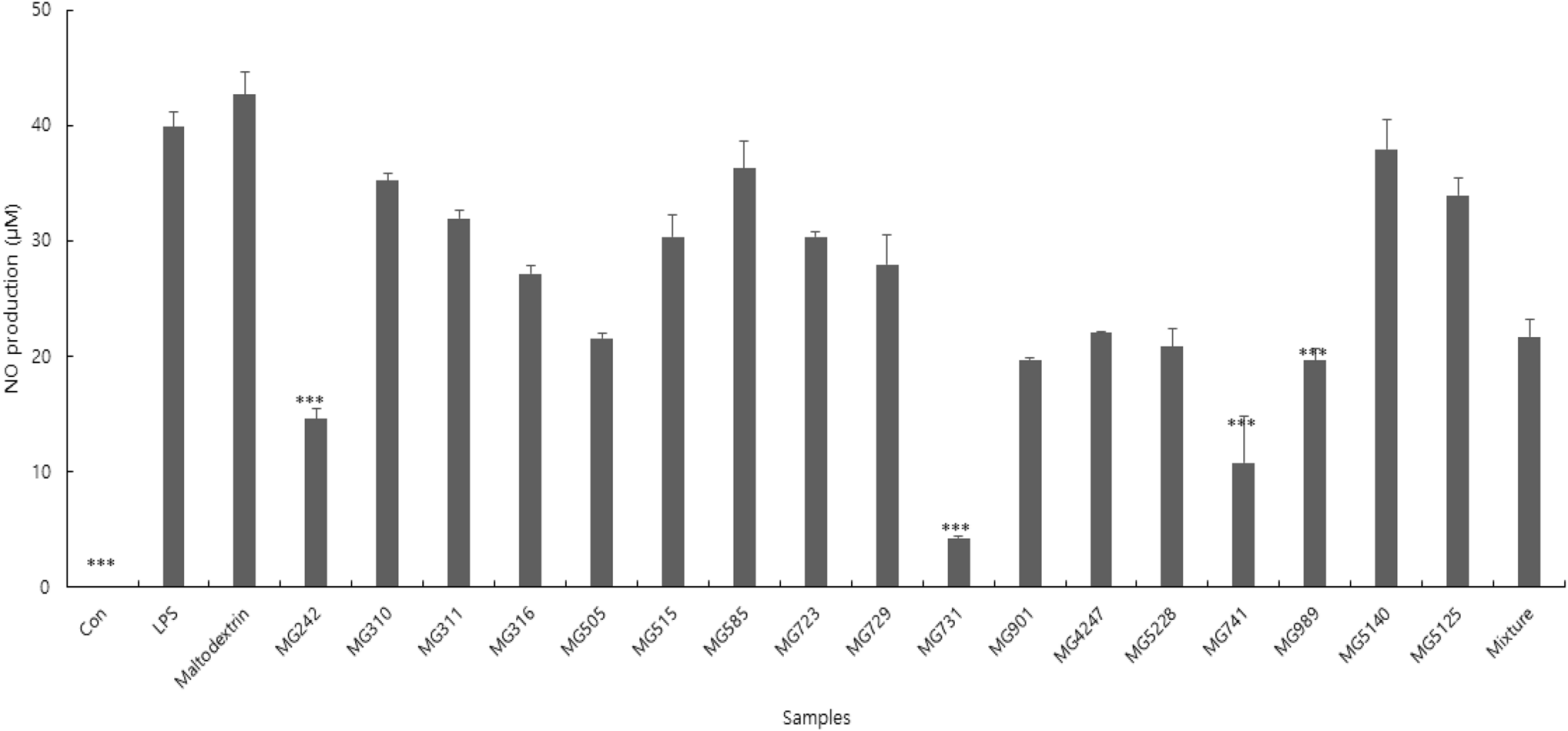
Inhibition of nitric oxide production in LPS-stimulated RAW264.7 cells treated with 18 samples (17 strains and mixture sample). The results are presented as mean ± standard deviation of three independent experiments (*n* = 3). ***P < .001, versus LPS

NO is a multi-functional mediator and plays a pivotal role in the immune response to inflammatory activity. The physiological or normal NO production in phagocytes is beneficial for the host defense against microorganisms, parasites, and tumor cells (Lin and Lin 1997; Korhonen et al. 2001). Results of the NO assay showed that the postbiotic samples showed a wide range of NO production levels (Fig. 1). This result indicated that the postbiotic samples might had different functional properties, even if they belong to the same species. Among the samples, *Bi. bifidum* MG731 exhibited the lowest NO production (4.28 µM) in LPS-stimulated cells, followed by *Bi. lactis* MG741 (10.80 µM), *L. salivarius* MG242 (14.60 µM), and *L. plantarum* MG989 (19.60 µM). To examine cytotoxicity, MTT assays were performed using the RAW 264.7 cells (data not shown). When treated with 18 samples, the cell viability ranged from 72.20 to 98.29%. Thus, the lowest NO production strains upregulated that cytokine expression and activated upstream pathways related to RAW 264.7 cells activation. Moreover, these strains were could be helpful in against invading pathogens via macrophage activation.

### Selected strains suppressed iNOS, COX-2, and TNF-α expression in LPS-stimulated RAW 264.7 cells

**Fig. 2:**
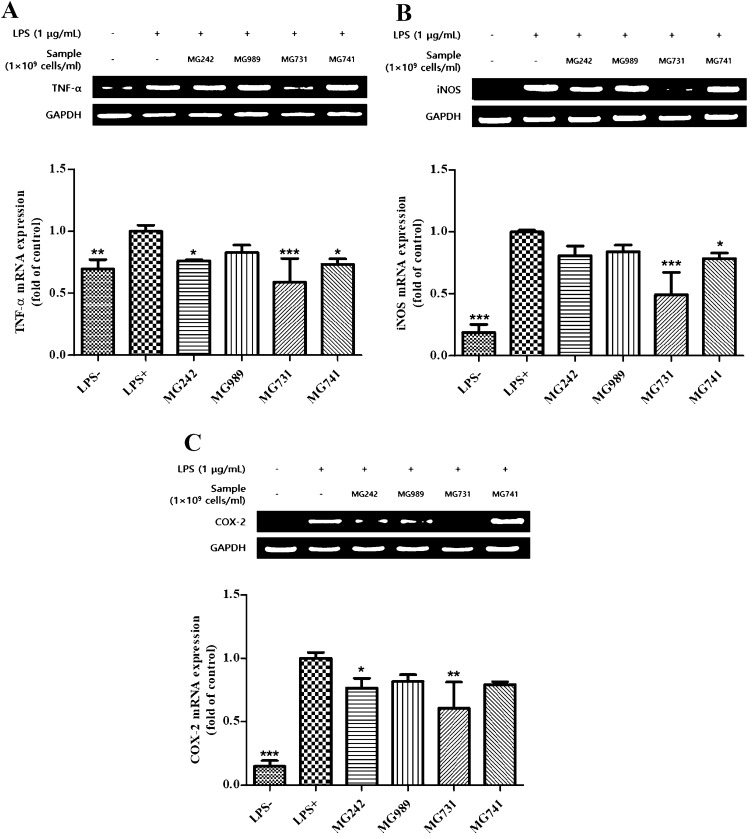
RT-PCR analysis of the mRNA expression of (**a**) TNF-α, (**b**) iNOS, and (C) COX-2. Values shown are mean ± standard deviation of three independent experiments. *P < .05; **P < .01; ***P < .001, versus LPS

NO and prostaglandins are modulated by distinct NOS and COX isoforms, respectively, and are important biomarkers in the inflammatory responses that induce pain, swelling, fever, and tenderness (Hu et al. 2008). Among the isoforms of these enzymes, iNOS and COX-2 are undetectable at the basal state. Using RT-PCR, we investigated whether selected strains (*L. salivarius* MG242, *L. plantarum* MG989, *Bi. bifidum* MG731, and *Bi. lactis* MG741) could regulate the expression of iNOS, COX-2, and TNF-α. As expected, treatment with LPS markedly increased the mRNA levels of iNOS, COX-2, and TNF- α, which were downregulated following treatment with the selected strains (Fig. 2a–c). The expression of the housekeeping gene GAPDH was not affected by the selected strains. These results indicated that treatment using selected strains might have inhibited the production of NO via the downregulation of iNOS and COX-2. Similarly, previous studies showed that treatment using heat-killed *Lactobacillus brevis* K65 decreases the production of NO in the RAW 264.7 cells upon LPS stimulation, which may be attributed to downregulated iNOS and COX-2 (Liu et al. 2016).

### Analysis of the SCFAs present in culture medium

Probiotics can produce various metabolites that antioxidant activity such as glutathione, butyrate, and folate. SCFAs, that are the major products of microbial fermentation of carbohydrates, can suppress the growth of pathogenic intestinal bacteria, and modulate lipid metabolism and the immune system (Rodrigues et al. 2016). In addition, SCFAs lower the intestinal pH and promote the bioavailability of minerals such as magnesium and calcium, thereby inhibiting the growth of harmful bacteria (Gullon et al. 2014).

The SCFA profile of the samples including the compounds and their concentrations is shown in Table 3. Acetic acid is the main SCFA produced by many intestinal bacteria in the human gut (Gullon et al. 2015), and was the most abundant SCFA found in this study. In addition, acetic acid is the metabolite generated the most during *Bifidobacterium* fermentation (Khodaei et al. 2016). The *Bifidobacterium* species showed a higher acetic acid production (356.0–4995.0 μg/g) than that of other species (316.0–1489.0 μg/g). These results suggest that the differences in the fermentation ability of specific strains may be due to the carbon chain length of the polysaccharides (Shalini et al. 2017). *Lactobacillus acidophilus* MG5228 showed a significantly higher propionic acid (19.9 μg/g) and butyric acid (80.7 μg/g) production than those of other strains. Propionic acid has been reported to reduce the activity of *Escherichia coli* and *Salmonella* spp. and has an anti-cholesterolemic effect (Khodaei et al. 2016). Butyrate is a SCFA produced by microbiota in the colon and distal small intestine from residual starch, dietary fiber, and low-digestible polysaccharides via fermentation (Kau et al. 2011). The MIYAIRI 588 strain of *Clostridium butyricum* is a butyrate-producing probiotic. It has recently been shown to induce antioxidases in rats with nonalcoholic fatty liver disease to suppress hepatic oxidative stress (Endo et al. 2013). The levels of antioxidant metabolites in the host can also be regulated via probiotic treatment. Furthermore, probiotics and their metabolites such as SCFAs play an essential role in human immune function (Sawicki et al. 2017).

**Table 3:** Short-chain fatty acid profile of 18 samples

Samples	AA (μg/g)	PA (μg/g)	BA (μg/g)	Total SCFA (μg/g)
*L. salivarius* MG242	1455.0 ± 6.8	0.95 ± 1.36	0.15 ± 0.05	1456.1 ± 8.2
*L. paracasei* MG310	867.0 ± 4.3	0.45 ± 0.06	0.05 ± 0.02	867.5 ± 4.4
*L. casei* MG311	802.0 ± 4.9	1.55 ± 0.27	2.05 ± 0.17	805.6 ± 5.3
*L. rhamnosus* MG316	1354.0 ± 9.4	1.15 ± 0.12	0.20 ± 0.08	1355.4 ± 9.6
*L. reuteri* MG505	1444.0 ± 9.2	0.85 ± 0.05	0.05 ± 0.02	1444.9 ± 9.3
*L. bulgaricus* MG515	316.0 ± 3.2	0.50 ± 0.01	0.65 ± 0.03	317.2 ± 3.2
*L. helveticus* MG585	1153.0 ± 9.3	0.95 ± 0.15	0.20 ± 0.01	1154.2 ± 9.5
*Bi. longum* MG723	3902.0 ± 14.9	0.60 ± 0.23	0.65 ± 0.03	3903.3 ± 15.2
*Bi. breve* MG729	356.0 ± 1.2	0.30 ± 0.07	0.45 ± 0.01	356.8 ± 1.3
*Bi. bifidum* MG731	4995.0 ± 22.6	1.55 ± 0.26	2.00 ± 0.35	4998.6 ± 23.2
*Bi. lactis* MG741	2610.0 ± 19.2	1.83 ± 0.03	2.05 ± 0.21	2613.9 ± 19.4
*L. fermentum* MG901	1294.0 ± 8.7	0.50 ± 0.02	0.00 ± 0.00	1294.5 ± 8.7
*L. plantarum* MG989	628.4 ± 2.7	0.88 ± 0.07	0.93 ± 0.18	630.2 ± 3.0
*L. gasseri* MG4247	428.0 ± 1.8	0.30 ± 0.02	0.25 ± 0.02	428.6 ± 1.8
*Lac. lactis* MG5125	1253.4 ± 4.3	1.03 ± 0.09	0.48 ± 0.08	1254.9 ± 4.5
*S. thermophilus* MG5140	971.7 ± 2.9	0.93 ± 0.04	0.23 ± 0.03	972.9 ± 3.0
*L. acidophilus* MG5228	1489.0 ± 2.6	19.90 ± 0.40	80.70 ± 3.63	1589.6 ± 6.6
Mixture	871.7 ± 2.7	3.60 ± 0.56	4.68 ± 0.34	880.0 ± 3.6

*AA* acetic acid; *PA* propionic acid; *BA* butyric acid

Data are presented as mean ± standard deviation (*n* = 3)

## Conclusion

The aim of this study was conducted with identifying superior postbiotic samples that showed desired antioxidant activity and production of SCFAs among 17 strains isolated from Korean individuals and fermented foods. We selected four strains that showed high antioxidant activity. Food products that comprise live probiotics as food ingredients require low temperature and anaerobic conditions to prolong shelf-life, which increases the cost of packaging and distribution. The use of heat-killed cells that still maintain their bioactivity can prolong the shelf-life and simplify the food processing steps of probiotic-containing foods owing to their high stability. The antioxidant and immune-modulating activities of selected strains in heat-killed forms show a strong potential for their usage in the manufacture of probiotic products.

## Data Availability

The authors declare that all data and materials support published claims and comply with feld standards.
